# Hydrogen storage and stability properties of Pd–Pt solid-solution nanoparticles revealed *via* atomic and electronic structure

**DOI:** 10.1038/s41598-017-14494-7

**Published:** 2017-11-03

**Authors:** Loku Singgappulige Rosantha Kumara, Osami Sakata, Hirokazu Kobayashi, Chulho Song, Shinji Kohara, Toshiaki Ina, Toshiki Yoshimoto, Satoru Yoshioka, Syo Matsumura, Hiroshi Kitagawa

**Affiliations:** 1 Synchrotron X-ray Station at SPring-8, Research Network and Facility Services Division, National Institute for Materials Science (NIMS), 1-1-1 Kouto, Sayo-cho, Sayo-gun, Hyogo, 679-5148 Japan; 2 Synchrotron X-ray Group, Research Center for Advanced Measurement and Characterization, NIMS, 1-1-1 Kouto, Sayo-cho, Sayo-gun, Hyogo, 679-5148 Japan; 3 Department of Materials Science and Engineering, School of Materials and Chemical Technology, Tokyo Institute of Technology, 4259-J3-16, Nagatsuta-cho, Midori-ku, Yokohama, 226-8502 Japan; 4 Division of Chemistry, Graduate School of Science, Kyoto University, Kitashirakawa Oiwake-cho, Sakyo-ku, Kyoto, 606-8502 Japan; 5 Research & Utilization Division, Japan Synchrotron Radiation Research Institute (JASRI), 1-1-1 Kouto, Sayo-cho, Sayo-gun, Hyogo, 679-5198 Japan; 6 Department of Applied Quantum Physics and Nuclear Engineering, Kyushu University, 744 Motooka, Nishi-ku, Fukuoka, 819-0395 Japan; 7 INAMORI Frontier Research Center, Kyushu University, 744 Motooka, Nishi-ku, Fukuoka, 819-0395 Japan; 8 Institute for Integrated Cell-Material Sciences (iCeMS), Kyoto University, Yoshida, Sakyo-ku, Kyoto, 606-8501 Japan

## Abstract

Bimetallic Pd_1−*x*_Pt_*x*_ solid-solution nanoparticles (NPs) display charging/discharging of hydrogen gas, which has relevance for fuel cell technologies; however, the constituent elements are immiscible in the bulk phase. We examined these material systems using high-energy synchrotron X-ray diffraction, X-ray absorption fine structure and hard X-ray photoelectron spectroscopy techniques. Recent studies have demonstrated the hydrogen storage properties and catalytic activities of Pd-Pt alloys; however, comprehensive details of their structural and electronic functionality at the atomic scale have yet to be reported. Three-dimensional atomic-scale structure results obtained from the pair distribution function (PDF) and reverse Monte Carlo (RMC) methods suggest the formation of a highly disordered structure with a high cavity-volume-fraction for low-Pt content NPs. The NP conduction band features, as extracted from X-ray absorption near-edge spectra at the Pd and Pt *L*_*III*_-edge, suggest that the Pd conduction band is filled by Pt valence electrons. This behaviour is consistent with observations of the hydrogen storage capacity of these NPs. The broadening of the valence band width and the down-shift of the *d*-band centre away from the Fermi level upon Pt substitution also provided evidence for enhanced stability of the hydride (Δ*H*) features of the Pd_1−*x*_Pt_*x*_ solid-solution NPs with a Pt content of 8-21 atomic percent.

## Introduction

Nanoparticles (NPs), particularly bimetallic NPs, have attracted much attention owing to their potential applications in numerous fields of science and technology. Compared with monometallic NPs, bimetallic NPs present various complex structural forms, such as core-shell^1–3^, multi-shell^4^ and ordered/random mixed solid-solution alloy NPs^5–7^. Furthermore, face-centred cubic (fcc) packed palladium and platinum noble metals can form a continuous solid-solution alloy for all compositions at high temperatures above 1043 K^8^. Density functional theory studies have shown that a Pd_1−*x*_Pt_*x*_ solid-solution phase is thermodynamically stable in the nanoparticle phase at 373 K, although Pd and Pt are immiscible in their bulk phases^9^. Such Pd_1−*x*_Pt_*x*_ solid-solution NPs systems could play an important role as effective catalysts^10–12^. Recently, we have fabricated Pd/Pt core/shell NPs and Pd_1−*x*_Pt_*x*_ solid-solution NPs to study their hydrogen-storage behaviour^1,5,13^. Palladium is well known for its hydrogen storage properties in both its bulk and NP forms^14^. Bulk Pt does not absorb hydrogen; however, Pt NPs with a diameter of 3.2 nm exhibit a hydrogen storage capability that increases with decreasing NP size^5,13,15^. The hydrogen-storage capacity of the Pd_1−*x*_Pt_*x*_ solid-solution NPs can be tuned by changing the composition of Pd and Pt. Notably, Pd_1−*x*_Pt_*x*_ solid-solution NPs with a Pt content of 8–21 atom % possess a higher hydrogen-storage capacity than that of Pd NPs^5^. Furthermore, these nanoparticles also possess a higher hydrogen-storage capacity than that of Pd/Pt core/shell NPs. According to computational investigations by Calvo and Balbuena, randomly-mixed-disordered and the ordered Pd-monolayers over a Pt system with the composition Pt_3_Pd_7_ are thermodynamically more favourable for the oxygen reduction reaction^16^. The electrocatalytic and hydrogen absorption/desorption properties of transition metals and their alloys strongly correlate with changes of the electronic and crystal structure of the catalyst^17–19^. Understanding the stable atomic-scale structures of Pd_1−*x*_Pt_*x*_ solid-solution NPs is crucial for enhancing their chemical and physical properties. The properties of these systems are highly dependent on particle size, composition, morphology and crystal structure^5,6,20,21^. Recent studies have postulated that the hydrogen dissociation of Pd_1−*x*_Pt_*x*_ solid-solution alloy is proportional to the hydrogen–metal bond strength and to the *d*-band centre position or the *d*-band states near the Fermi level^22,23^. Furthermore, an increase of the enthalpy of metal hydride formation, $i.e.$ a decrease of the exothermic heat has been reported, implying a decrease of the hydride stability and a decrease in the chemical bonds strength between metal and hydrogen atoms^13,24^. Moysan *et al*. have reported that, upon Pt substitution of Pd_1−*x*_Pt_*x*_ solid-solution alloys, the enthalpy of the hydride formation increases and the hydride stability decreases owing to considerable broadening of the valence band^25^.

In this study, we investigate the average crystallographic structure and local structure of Pd_1−*x*_Pt_*x*_ solid-solution NPs (for 0 $\le$ $x$ $\le$ 0.5) by means of high-energy X-ray diffraction coupled with atomic pair distribution function analysis and reverse Monte Carlo (RMC) modelling techniques. Extended X-ray absorption fine structure (EXAFS) analysis was also applied to investigate the local atomic structure around Pd and Pt atoms in Pd_1−*x*_Pt_*x*_ solid-solution NPs. To reveal correlations between properties of the electronic structure, such as the unoccupied electronic states and their density of states (DOS), and the hydrogen storage capacity and stability of the Pd_1−*x*_Pt_*x*_ solid-solution NPs, we used hard X-ray photoelectron spectroscopy (HAXPES) and X-ray absorption near-edge spectroscopy (XANES).

## Results

### X-ray scattering characterization

**Figure 1:**
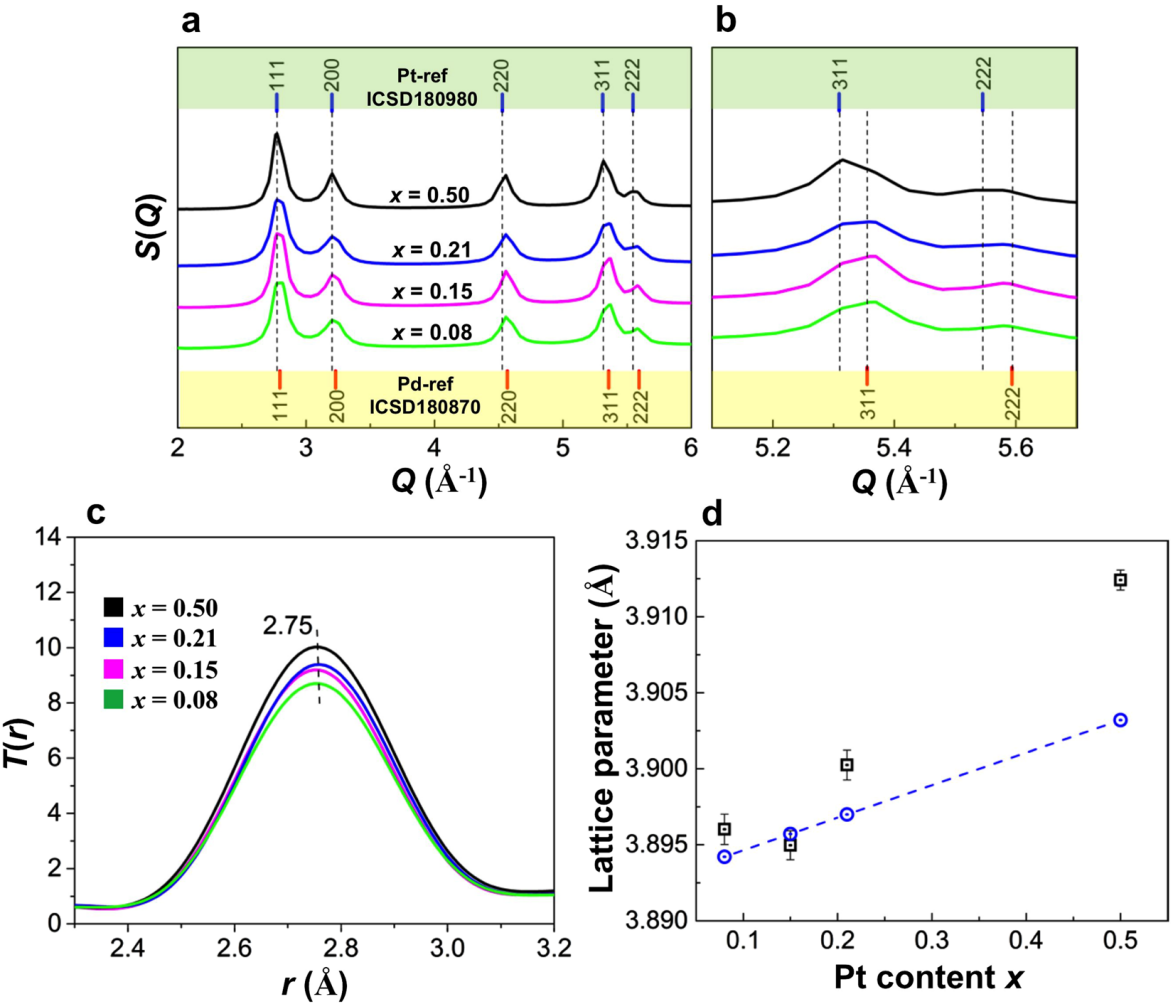
Experimental high-energy XRD data and lattice parameters for Pd_1−*x*_Pt_*x*_ solid solution nanoparticles. (**a**) The total structure factor, *S*(*Q*), for Pd_1−*x*_Pt_*x*_ solid solution NPs and (**b**) extended view of 311 and 222 peaks. (**c**) Total correlation function, $T(r)$, for Pd_1-__*x*_Pt_*x*_ solid solution NPs. (**d**) Composition dependence of the lattice parameters of Pd_1−*x*_Pt_*x*_ solid solution NPs obtained by pair distribution function (PDF) fittings (black square) and calculated by Vegard’s law (blue circle).

As we have previously reported, high-resolution transmission electron microscope (TEM) images and energy-dispersive X-ray spectroscopy (EDS) spectra of the Pd_1−*x*_Pt_*x*_ solid-solution NPs have revealed that Pd and Pt are homogeneously mixed at the atomic level by the process of hydrogen absorption/desorption (PHAD) at 373 K, which is a trigger for formation of Pd/Pt core/shell NPs^5,11,26,27^. Using the results of the TEM measurements, we determined the mean diameters of the Pd_1−*x*_Pt_*x*_ solid-solution NPs for the compositions where $x$ = 0.08, 0.15, 0.21 and 0.5 to be 6.7 $\pm$ 0.9, 7.4 $\pm$ 0.9, 8.1 $\pm$ 1.0 and 11.2 $\pm$ 1.7 nm, respectively (see Table 1)^5^. Figure 1a shows a comparison of the experimental total structure factors, *S*(*Q*), of the Pd_1−*x*_Pt_*x*_ solid-solution NPs with standard diffraction peak positions of Pd (ICSD 180980, fcc lattice constant 3.8911 Å) and Pt (ICSD 180870, fcc lattice constant 3.925 Å)^28^. Herein, the *S*(*Q*) patterns of the Pd_1−*x*_Pt_*x*_ solid-solution NPs are intermediate between the diffraction patterns for the two bulk metals, demonstrating the formation of Pd_1−*x*_Pt_*x*_ solid-solution alloys with a single fcc lattice^29,30^. Figure 1a and b show the composition dependence of the experimental *S*(*Q*) values of the Pd_1−*x*_Pt_*x*_ solid-solution NPs for $x$ = 0.08, 0.15, 0.21 and 0.5. All of the *S*(*Q*) of the Pd_1−*x*_Pt_*x*_ solid-solution NPs exhibit the characteristic 111, 200, 220, 311 and 222 peaks of the fcc structure^20^. With increasing Pt content $x$, the extended view of the 311 peak profile of the Pd_1−*x*_Pt_*x*_ solid-solution NPs exhibits a small shift of the peak to lower $Q$ with respect to that of bulk Pd (see Fig. 1b). All the NP spectra feature broad asymmetric peaks owing to nano-sizing effects. Conversely, the diffraction pattern of the Pd/Pt core/shell NPs is similar to that of the Pd NP (Pd-core) structure for Pt contents less than 0.21, as shown in Supplementary Fig. S1a. The Fourier transform (FT) of the *S*(*Q*) (Fig. 1c), the total correlation function $T(r)$, indicates that the similar peak position of M–M (where M = Pd, Pt) interatomic distance at ~2.75 Å for all Pd_1−*x*_Pt_*x*_ solid-solution NPs reflecting the similarity of the basic structure. In particular, the $T(r)$ features become broadened out and peak intensity is increased with increase of Pt content as well as NP size due to the finite size effects. Furthermore, the first three peaks in the total correlation function $T(r)$, are observed at distances of 2.74, 3.88 and 4.76 Å for bulk Pd and 2.77, 3.91 and 4.80 Å for bulk Pt, respectively (see Supplementary Fig. S1b). These local structure results were consistent with previously-reported PDF data for Pd-Pt alloy NPs^31^. Figure 1d shows the fcc lattice parameters of the NPs, which were obtained by comparing the experimental and calculated reduced PDF *G*(*r*) (see Supplementary Fig. S2 and Table 1) with the use of the PDFgui program^32,33^. According to these results, the lattice parameters of the Pd_1−*x*_Pt_*x*_ solid-solution NPs decrease with decreasing Pt content $x$. As Fig. 1d shows, Vegard’s law is not strictly obeyed by the Pd_1−*x*_Pt_*x*_ solid-solution NPs. According to the Vegard’s law^34^

1 $${a}_{PdPt}=\mathrm{(1}-x){a}_{Pd}+x{a}_{Pt},$$

where *a*_*PdPt*_ is the lattice parameter of the Pd_1−*x*_Pt_*x*_ solid-solution alloy; ${a}_{Pd}$ and ${a}_{Pt}$ are the lattice parameters 3.8925^35^ and 3.9139 Å^36^ of Pd and Pt NPs in size 10 nm, respectively; and $x$ is the molar fraction of Pt. The composition dependence of the lattice parameter for the Pd_1−*x*_Pt_*x*_ solid-solution NPs for $x$ = 0.08, 0.21 and 0.5 exhibits a significant positive deviation from the linear relation predicted by Vegard’s law, indicating that the solid-solution NPs are under significant tensile stress^7^. Conversely, the lattice parameters of the NPs with $x$ = 0.15 exhibit negligible negative deviation from Vegard’s law for Δ*a* = −0.0007 Å due to the weak compressive stress. An apparent negative deviation from Vegard’s law has been reported for a bulk Pd_1−*x*_Pt_*x*_ (0 $\le$ $x$ $\le$ 0.5) solid-solution alloy, indicating that the lattice is less expanded under strong compressive stress and electronegativity difference between the metal atoms^25^.

**Table 1:** Atomic ratio, size, lattice parameters and hydrogen pressure-composition isotherms data of Pd_1−*x*_Pt_*x*_ solid solution nanoparticles.

Sample	Atomic ratio Pd:Pt	Size (nm)	Lattice Parameter (Å)	H/M (wt. %)	${\boldsymbol{\Delta }}{\boldsymbol{H}}$ (KJ/mol H_2_)
Pd_0.92_Pt_0.08_	92:8	6.7 $\pm$ 0.9	3.8960 $\pm$ 0.0010	0.41	$-$30.0
Pd_0.85_Pt_0.15_	85:15	7.4 $\pm$ 0.9	3.8949 $\pm$ 0.0010	0.40	$-$24.5
Pd_0.79_Pt_0.21_	79:21	8.1 $\pm$ 1.0	3.9002 $\pm$ 0.0010	0.32	$-$25.0
Pd_0.5_Pt_0.5_	50:50	11.2 $\pm$ 1.7	3.9124 $\pm$ 0.0007	0.16	$-$18.0

The atomic ratio of all NPs were estimated by ICP-MS analysis. The size of the NPs was determined from TEM images. Lattice parameters were obtained from PDF fittings based on high-energy XRD data of NPs. Hydrogen storage capacity (H/M) and stability (Δ*H*) of NPs were measured with a PCT apparatus.

### Reverse Monte Carlo modelling

**Figure 2:**
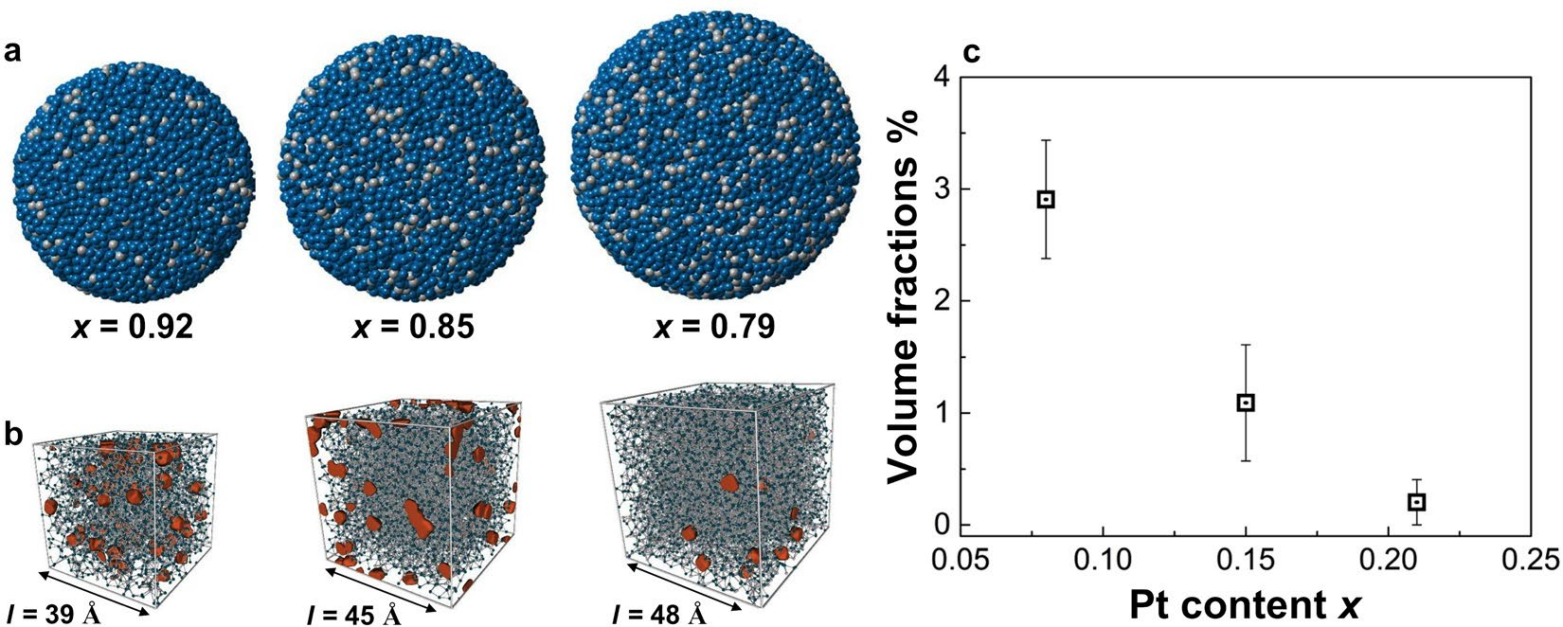
The 3D reverse Monte Carlo (RMC) models and cavity sites. (**a**) Atomic RMC configuration of Pd_1−*x*_Pt_*x*_ solid solution NPs (Pd in cyan and Pt in grey), (**b**) cavities within a cubic volume extracted from the RMC model of the NPs (cavity sites in orange) and (**c**) volume fraction of the cavities as a function of Pt content $x$.

Three-dimensional (3D) atomic-scale structural models of the Pd_1−*x*_Pt_*x*_ solid-solution NPs were constructed with the use of RMC modelling based on high-energy X-ray diffraction data. Notably, RMC modelling is suitable for studying isolated and finite-sized spherical NPs without structural periodicity or uniformity^37^. The RMC model *S*(*Q*) and experimental *S*(*Q*) data sets for the Pd_1−*x*_Pt_*x*_ solid-solution NPs ($x$ = 0.08, 0.15, 0.21) are shown in Supplementary Fig. S3. Herein, the RMC configuration models of the NPs show good agreement with the experimental *S*(*Q*). As shown in Fig. 2a, RMC configuration models indicate the highly-disordered structure and spherical shape of the Pd_1−*x*_Pt_*x*_ solid-solution NPs. The RMC models for the $x$ = 0.08, 0.15 and 0.21 solid-solution NPs exhibit a good degree of chemical alloying without the manifestion of onion or core/shell type configurations. Figure 2b shows the distribution of cavities within the Pd_0.92_Pt_0.08_, Pd_0.85_Pt_0.15_ and Pd_0.79_Pt_0.21_ solid-solution NPs in a cubic volume with a length of 39, 45 and 48 Å, respectively. Full details of the cavity analysis procedure have been described elsewhere^38,39^. Interestingly, the high-Pd-content Pd_0.92_Pt_0.08_ solid-solution NPs exhibit a large volume fraction (2.9%) of cavities. In contrast, we observe relatively low cavity volume of 0.2% and 1.1% for the Pd_0.79_Pt_0.21_ NPs and Pd_0.85_Pt_0.15_ solid-solution NPs, respectively (see Fig. 2c). These results indicate that the volume fraction of cavities in the Pd_1−*x*_Pt_*x*_ solid-solution NPs decreases with increasing Pt content $x$.

### XANES & EXAFS characterization

**Figure 3:**
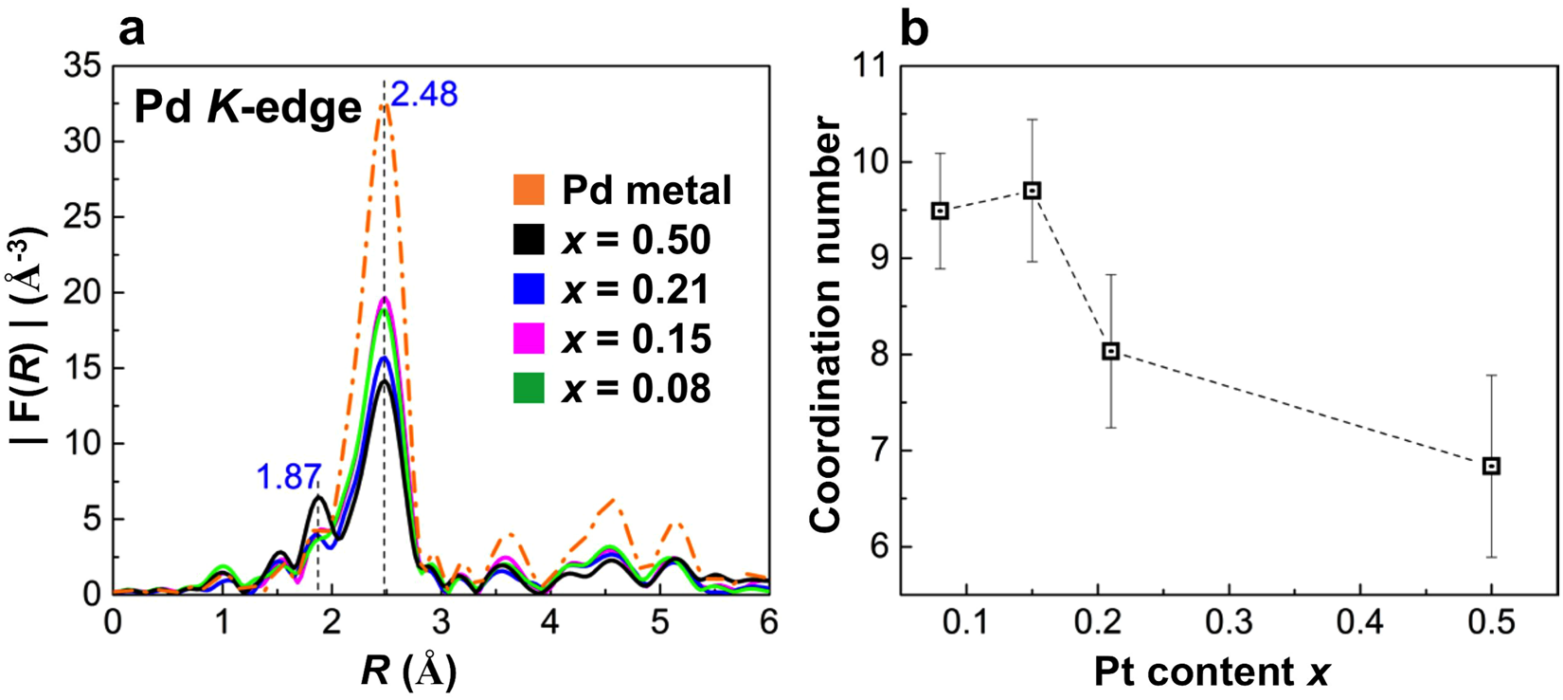
Fourier transform (FT) of the Pd *K*-edge extended X-ray absorption fine structure (EXAFS) and first shell coordination number analysis. (**a**) Fourier transforms of Pd *K*-edge EXAFS spectra (phase shift uncorrected) of Pd_1−*x*_Pt_*x*_ solid solution NPs for $0\le x\le 0.5$ and Pd metal foil. (**b**) First-nearest-neighbour coordination number $versus$ Pt content $x$.

**Figure 4:**
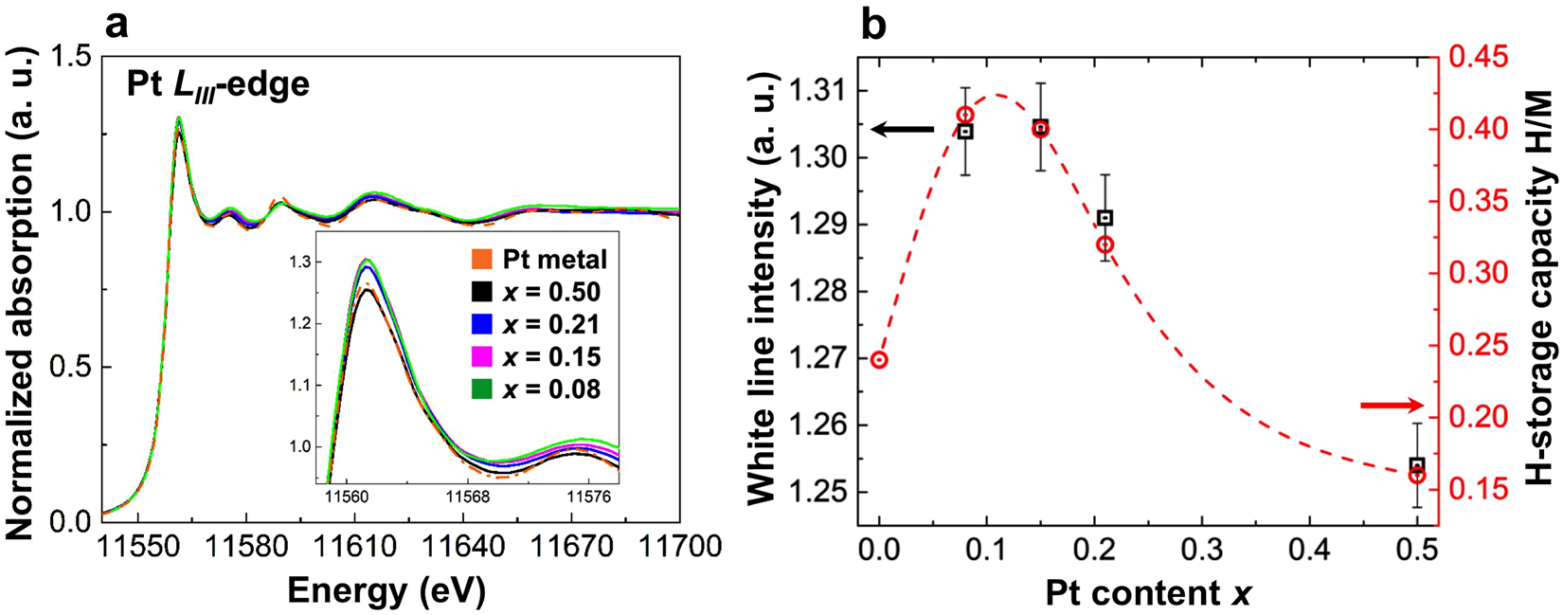
Pt ${L}_{III}$-edge X-ray absorption near-edge spectroscopy (XANES) spectra and correlation between white line intensity and hydrogen storage capacity. (**a**) Pt ${L}_{III}$-edge XANES spectra of Pd_1−*x*_Pt_*x*_ solid solution NPs and Pt metal foil and (**b**) the white line intensity (black square) and hydrogen storage capacity (red circle) as a function of Pt content $x$.

The X-ray absorption near-edge structure (XANES) spectra at the Pd $K$-edge for the NP samples and bulk Pd metal foil are characterized by two maxima appearing at 24363.0 and 24386.5 eV (see Supplementary Fig. S4)^40,41^. The positions of these two peaks for the Pd_0.5_Pt_0.5_ solid-solution NPs are almost same as those for bulk Pd, while the peaks are broadened owing to size effects. However, the second peak of the Pd_0.85_Pt_0.15_ and Pd_0.92_Pt_0.08_ solid-solution NPs exhibits peak narrowing as well as a small shift to higher energy (~2 eV) compared with the second peak of the Pd_0.5_Pt_0.5_ solid-solution NPs. The XANES spectra at the Pd ${L}_{III}$-edge exhibit a significant difference in the integrated intensity and the peak maxima at the main absorption edge ($i.e.$ the “white line”) of the Pd_0.92_Pt_0.08_, Pd_0.85_Pt_0.15_ and Pd_0.79_Pt_0.21_ solid-solution NPs compared with those of the Pd NPs and Pd_0.5_Pt_0.5_ solid-solution NPs (see Supplementary Fig. S5a–c and Note S6). The FT of the extended X-ray absorption fine structure (EXAFS) spectra (phase shift uncorrected) at the Pd $K$-edge of the Pd_1−*x*_Pt_*x*_ solid-solution NPs for 0 $\le$ $x$ $\le$ 0.5 and the Pd metal foil are shown in Fig. 3a. For the Pd_1−*x*_Pt_*x*_ solid-solution NPs, the main peak at 2.48 Å is assigned to the Pd–Pd correlation (2.74 Å) and a large shoulder peak is observed at 1.87 Å^42,43^. The amplitude of this Pd–Pd peak decreases simultaneously with increasing Pt content $x$, while the amplitude of a smaller peak at 1.87 Å increases. Thus, the peak at 1.87 Å can likely be attributed to the Pd–Pt correlation of the Pd_1−*x*_Pt_*x*_ solid-solution NPs. The results of the refined parameter values extracted $via$ EXAFS analysis of the Pd $K$-edge EXAFS data of the Pd_1−*x*_Pt_*x*_ solid-solution NPs are listed in Supplementary Table S1. The first shell analysis reveals that the Pd–Pd bond distance (2.74 Å) of the Pd_1−*x*_Pt_*x*_ solid-solution NPs is similar to that of the Pd bulk, and the first-nearest-neighbour coordination number around the absorbing Pd atom gradually increases with decreasing Pt content $x$ (see Fig. 3b and Supplementary Table S1). However, the coordination number differences of the Pd_1−*x*_Pt_*x*_ solid-solution NPs for $0.08\le x\le 0.5$ cannot be explained by the first shell Debye–Waller factor. Furthermore, the discrepancies of the Pd–Pd bond distance and coordination number between the Pd NPs and Pd bulk may be related to the contribution of the large number of surface atoms present in the NPs^44^. The XANES and EXAFS measurements of the Pt ${L}_{III}$-edge were performed on the Pd_1−*x*_Pt_*x*_ solid-solution NPs samples as well as a Pt standard metal foil. As shown in Fig. 4a, the Pt ${L}_{III}$-edge XANES spectra of the Pd_1−*x*_Pt_*x*_ solid-solution NPs correspond well with the spectrum of elemental Pt. The normalized white line intensity variation at 11561.3 eV (see Fig. 4a inset) of the Pd_1−*x*_Pt_*x*_ solid-solution NPs is consistent with the increasing Pt content $x$^25^. The FTs of the Pt ${L}_{III}$-edge EXAFS spectra (phase shift uncorrected) of Pt metal foil in Supplementary Fig. S6 show a shoulder peak at 2.11 Å and a main peak at 2.63 Å owing to a phase shift induced by the potential of the absorbing and scattering atoms and the terminal effect in the FT^42,43^. For the Pd_1−*x*_Pt_*x*_ solid-solution NPs of $x\le 0.21$, the Pt–Pt bond distance increases and the two peaks are clearly separated, as is seen for the Pt metal foil. This can be attributed to the phase shift of the Pt–Pd bond caused by interference between the Pd and Pt atoms. Note that the Pd_0.92_Pt_0.08_ solid-solution NPs exhibit shortening of the Pt–Pt bond distance (see Supplementary Fig. S6) compared with the Pd_0.85_Pt_0.15_ and Pd_0.79_Pt_0.21_ solid-solution NPs, which might originate from the formation of an ionic bond, as has been suggested by Matsubayashi *et al*.^43^.

### Valence-band spectra characterization

**Figure 5:**
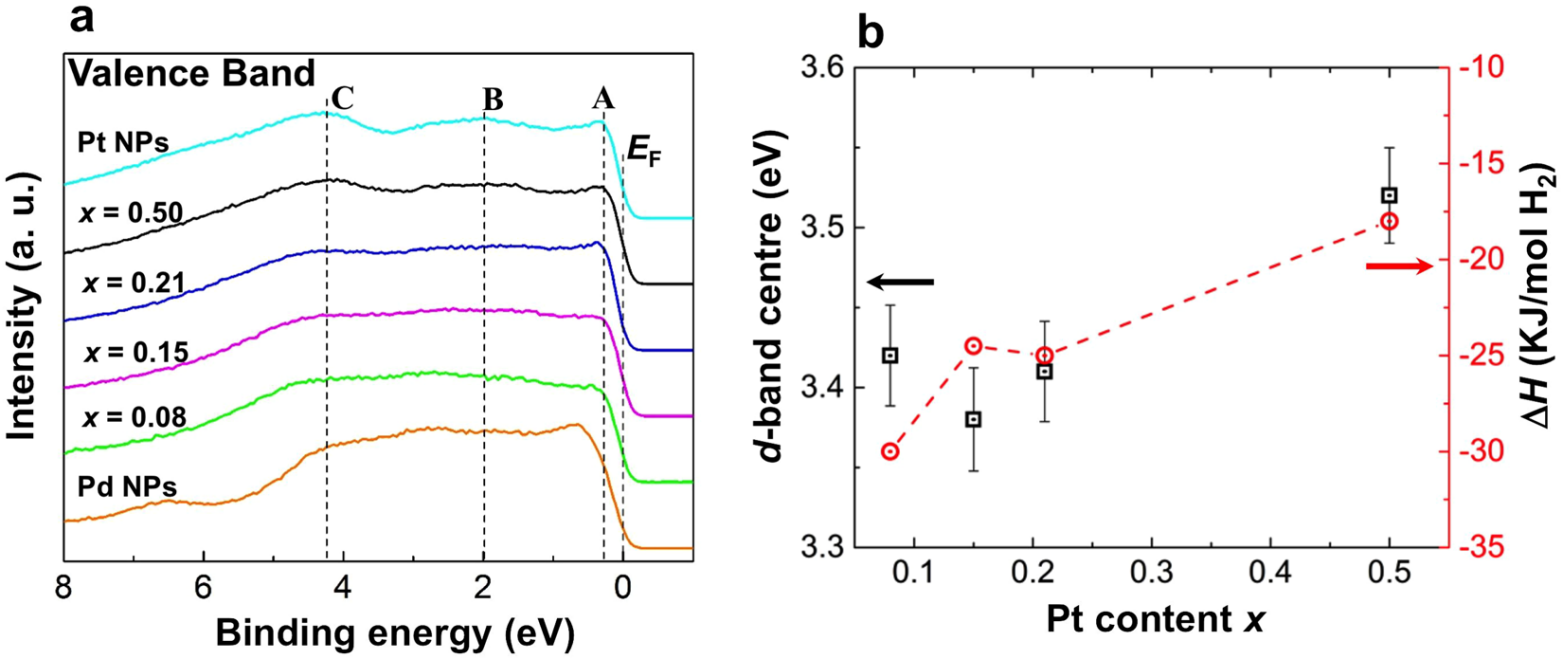
Hard X-ray photoelectron spectroscopy (HAXPES) valence-band (VB) spectral analysis and the stability of the hydride. (**a**) VB spectra of Pd_1−*x*_Pt_*x*_ solid solution NPs for $0.08\le x\le 0.5$, Pd and Pt NPs. (**b**) Correlation between *d*-band centre (black square) and stability of the hydride (Δ*H*) (red circle) as a function of Pt content $x$.

Hard X-ray photoelectron spectroscopy (HAXPES) valence-band (VB) spectra of the Pd_1−*x*_Pt_*x*_ solid-solution NPs for $0.08\le x\le 0.5$, Pd NPs and Pt NPs are presented in Fig. 5a. The VB spectra were measured at a photon energy 5.95 keV, and the Shirley background was subtracted across the whole region. The VB spectra for all Pd_1−*x*_Pt_*x*_ solid-solution NPs are dominated by the Pt 5$d$ band states at the Fermi level, owing to the larger photoionization cross-section of Pt 5$d$ (137 kb) compared with that of Pd 4$d$ (78.4 kb) at 6 keV^45^. Broadening of the NP VB spectra gradually increases with increasing Pt content $x$. In addition, the VB spectra of the Pd_1−*x*_Pt_*x*_ solid-solution NPs exhibit features (marked as A, B and C in Fig. 5a) that are identical to those of Pt NPs^25^. The observed VB spectra in Fig. 5a approximately correspond to the electron-occupied $d$ orbital of the metal (*d*-band density of states) because the kinetic energy of the excited electrons in the HAXPES is sufficiently high to avoid any resonance from unoccupied electron states^46^. As shown in Fig. 5b, we evaluated the *d*-band centre using $\int DOS(E)EdE/\int DOS(E)dE$, where $DOS(E)$ represents the DOS of the occupied $d$-states and $E$ is the energy of the state^47^.

## Discussion

**Figure 6:**
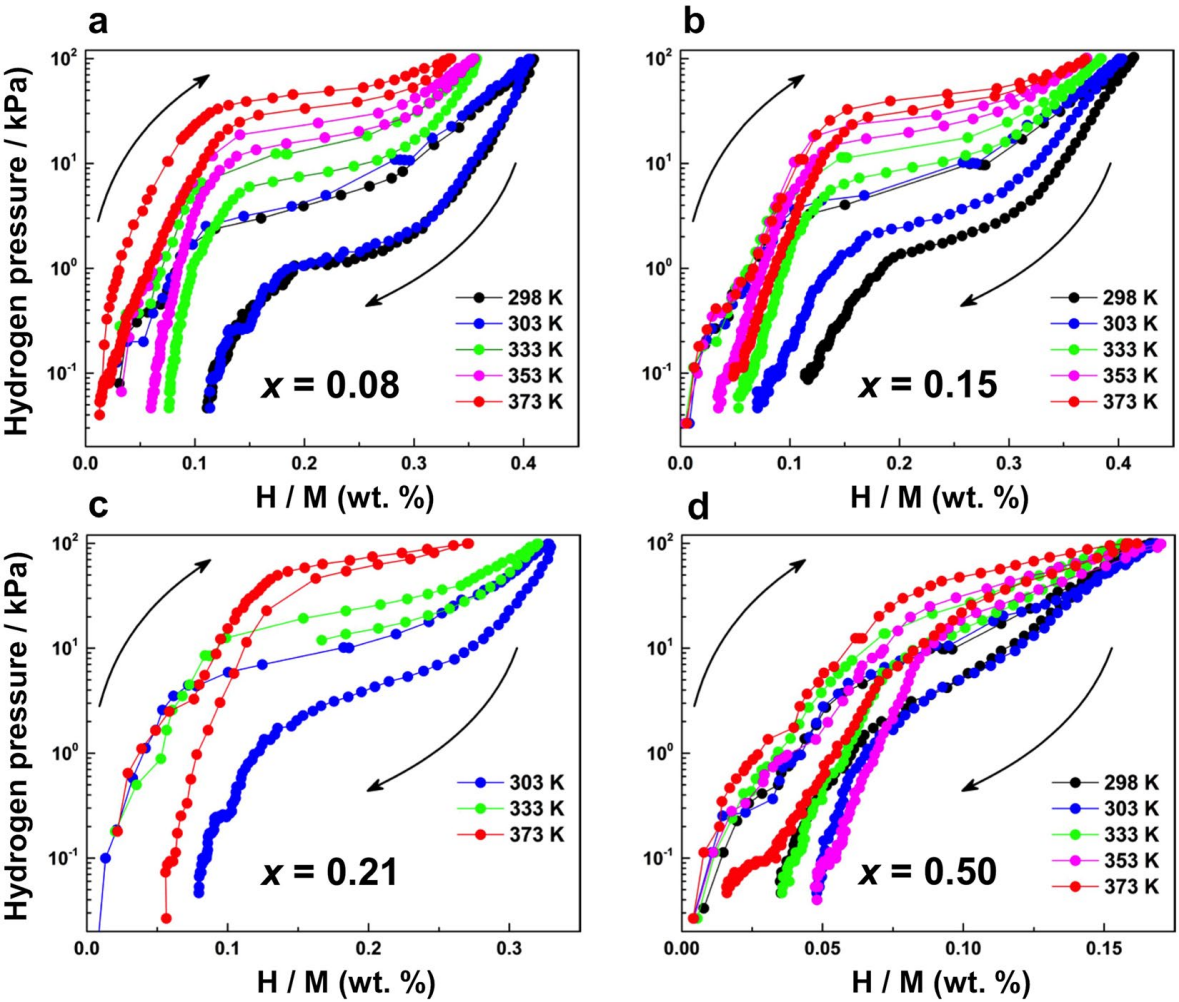
Pressure-composition-temperature (PCT) isotherms measurement. Hydrogen PCT isotherms of (**a**) Pd_0.92_Pt_0.08_, (**b**) Pd_0.85_Pt_0.15_, (**c**) Pd_0.79_Pt_0.21_ and (**d**) Pd_0.5_Pt_0.5_ solid-solution NPs. PCT isotherms were measured according to the direction of arrows. H/M shows the number of hydrogen atoms divided by the total number of metal atoms.

The Pd_1−*x*_Pt_*x*_ solid-solution NPs were prepared by PHAD at 373 K. The 3D atomic-scale structures of the Pd_1−*x*_Pt_*x*_ solid-solution NPs were studied by high-energy XRD coupled with PDF and RMC methods, together with EXAFS, a sensitive probe for the average distribution of atomic neighbours ($i.e.$, local structure). Furthermore, the electronic structures of the NPs were investigated with the use of XANES and HAXPES. The fcc lattice parameters of the Pd_1−*x*_Pt_*x*_ solid-solution NPs, evaluated from fitting the experimental PDF values with the calculated models, are found to decrease with decreasing Pt content $x$. However, we observe that the Pd_1−*x*_Pt_*x*_ solid-solution NPs do not follow Vegard’s law, as illustrated in Fig. 1d. The observed deviation of the lattice parameters from Vegard’s law is assumed to be associated with various physical factors such as the relative atomic sizes of the Pd and Pt metals, the relative volume per valence electron in the crystals of the pure metals as well as electro-chemical differences between the Pd and Pt metal composites in the alloyed NPs^25,34^. However, we also observe that the unit cell volume of the NPs increases with the addition of Pt atoms. Geometric effects such as the metal–metal bond distance, unit cell volume and surface area cannot explain the corresponding hydrogen absorption properties of Pd_1−*x*_Pt_*x*_ solid-solution NPs. Furthermore, the 3D RMC configuration models of the Pd_0.92_Pt_0.08_, Pd_0.85_Pt_0.15_ and Pd_0.79_Pt_0.21_ solid-solution NPs indicate highly disordered structures (see Fig. 2a). It is well-known that, in metals, hydrogen has a high probability of becoming trapped in cavities ($i.e.$, vacancies)^48^. Figure 2b shows the cavity volumes, or possible hydrogen trapping sites, together with the atomic configurations of the Pd_1−*x*_Pt_*x*_ solid-solution NPs. Note that the Pd_0.92_Pt_0.08_ solid-solution NPs have the highest cavity volume (see Fig. 2c), which is consistent with the previously reported hydrogen storage properties of Pd_1−*x*_Pt_*x*_ solid-solution NPs^5^. However, the Pd_1−*x*_Pt_*x*_ solid-solution NP structure for the various compositions is maintained to preserve the local order, and disorder is introduced with the substitution of Pt metal atoms because these atoms are packed together as closely as possible. Furthermore, the above-mentioned change of the atomic arrangement as a function of increasing Pt content $x$ suggests a random distribution of substitutional Pt atoms (see Fig. 2a), which induce a decrease in the number of cavity sites likely to be occupied by hydrogen atoms, as demonstrated in Fig. 2c. As Fig. 3b shows, the first shell coordination number of Pd increases with decreasing Pt content $x$, but remains below 12 for all compositions, while the lattice parameters of the NPs decrease (see Fig. 1d and Table 1). This means that the Pd atoms are more closely packed in the high-Pd-content solid-solution NPs. According to these results, the higher hydrogen-storage capacity of the Pd_0.92_Pt_0.08_ (or Pd_1−*x*_Pt_*x*_ with $0.08\le x\le 0.21$) solid-solution NPs (see Fig. 6) might also be caused by the lower lattice parameters that accompany high Pd–Pd coordination numbers (see Figs. 1d and 3b, respectively). As shown in Fig. 6, the plateau-like region (miscible state in the phase transition from the solid-solution ($\alpha$) phase and hydrogen to the hydride ($\beta$) phases) of the pressure-composition (PC) isotherm of Pd_0.92_Pt_0.08_ solid-solution NPs becomes more clear in comparison with that of high Pt content Pd_0.5_Pt_0.5_ solid-solution NPs^5,13^. On the other hand, the pressure gap widths in the miscible region for all solid-solution NPs becomes smaller with increasing temperature. This results support that largely enhanced hydrogen storage capacity of Pd_0.92_Pt_0.08_ solid-solution NPs where Pd and Pt atoms are mixed homogeneously^5^.

The XANES measurements at the Pt ${L}_{III}$ absorption edge white line intensity probe *d*-band vacancies in the conduction band (CB) corresponding to the electronic transition from Pt $2{p}_{\mathrm{3/2}}$ to the unoccupied Pt $5d$-states^25^. When the Pt content $x$ of the Pd_1−*x*_Pt_*x*_ solid-solution NPs increases, the Pt ${L}_{III}$-edge white line intensity decreases (see Fig. 4a inset and 4b), indicating the filling of holes in the CB. Therefore the hydrogen solubility of Pd_1−*x*_Pt_*x*_ solid-solution NPs markedly decreases with increasing Pt content owing to the lower number of holes in the CB (see Fig. 4b and Table 1). We also confirmed the filling of the Pd $4d$ CB by Pt valence electrons from Pd ${L}_{III}$-edge XANES spectra (see Supplementary Fig. S5a). The integrated intensity of the Pd ${L}_{III}$-edge white line intensity, which can be attributed to the number of $4d$-band vacancies (holes) ($i.e.$ unoccupied states of the final $4d$-band)^49^, correlates with the H-storage capacity, H/M (see Supplementary Fig. S5b and Fig. 6). Notably the white line peaks of the Pd_0.92_Pt_0.08_, Pd_0.85_Pt_0.15_ and Pd_0.79_Pt_0.21_ solid-solution NPs occur at lower energies than those of the Pd NPs and Pd_0.5_Pt_0.5_ solid-solution NPs. These peaks, observed at a relatively low energy, indicate that the excitation energy from the Pd $2{p}_{\mathrm{3/2}}$ band to the unoccupied $4d$ band is small, which also implies a greater number of $4d$ band holes in the Pd_0.92_Pt_0.08_, Pd_0.85_Pt_0.15_ and Pd_0.79_Pt_0.21_ solid-solution NPs compared with those in the Pd NPs and Pd_0.5_Pt_0.5_ solid-solution NPs (see Supplementary Fig. S5c).

According to the VB HAXPES results, the enhancement of the hydrogen absorption and solubility properties of the Pd_1−*x*_Pt_*x*_ solid-solution NPs can be explained by the large broadening of the VB owing to Pt atom substitution (see Fig. 5a). As can be seen in Fig. 5a, the VB spectra of the Pd_1−*x*_Pt_*x*_ solid-solution NPs are narrower than those of the Pt NPs but are considerably broader than those of the Pd NPs. However, the *d*-band centre of the Pd_1−*x*_Pt_*x*_ solid-solution NPs decreases with decreasing Pt content *x* (see Fig. 5b). The position of the *d*-band centre should be related to the strain and electronic coupling between Pd and Pt atoms^50^, where strain tends to down-shift the energy of the *d*-band centre away from the Fermi level owing to weak bonding with adsorbates such as hydrogen. The effect of the electronic coupling is also a key parameter for the aforementioned tendency owing to a change in the DOS near the Fermi level. As can be seen in Fig. 5b, the stability of the hydride is consistent with our experimental results of the *d*-band centre. This result can be explained as follows: when the *d*-band centre is close to the Fermi level, antibonding states can be shifted well above the *d*-band state and become unoccupied in the metal, thereby increasing the bond strength of the metal hydride (Pd–H and Pt–H)^51^. Herein, the stability of the hydride was estimated from the thermodynamic parameters of exothermic heat (${\rm{\Delta }}{H}_{\alpha \to \beta }$) and entropy loss (${\rm{\Delta }}{S}_{\alpha \to \beta }$), obtained from the temperature dependence of the equilibrium pressure of the pressure-composition (PC) isotherms for the phase transition $\alpha$ to $\beta$ (see Fig. 6)^5^. The ${\rm{\Delta }}{H}_{\alpha \to \beta }$ and ${\rm{\Delta }}{S}_{\alpha \to \beta }$ are given by the Van’t Hoff plots represented by^13^:

2 $$ln\frac{{P}_{abs}}{{P}_{0}}=\frac{{\rm{\Delta }}{H}_{\alpha \to \beta }}{RT}-\frac{{\rm{\Delta }}{S}_{\alpha \to \beta }}{R},$$

where ${P}_{abs}$ and ${P}_{0}$ are the equilibrium hydrogen pressure and the standard hydrogen pressure, respectively. According to the Griessen and Driessen model reported by Moysan *et al*.^25^, the exothermic heat (${\rm{\Delta }}H$) of hydride formation decreases and the hydride stability increases with decreasing Pt content $x$. As shown in a previous study by Yamauchi *et al*.^13^, ${\rm{\Delta }}H$ is related to the stability of the chemical bond between Pd and hydrogen atoms in the $\beta$ phase. Therefore, the increase of hydrogen stability (decrease of ${\rm{\Delta }}H$ value) of Pd_1−*x*_Pt_*x*_ ($0.08\le x\le 0.21$) solid-solution NPs can be explained by the movement of the *d*-band centre nearer to the Fermi level, perhaps while retaining the strong bond strength of hydride formation with decreasing Pt content $x$ (see Fig. 5b and Table 1). The atomic and electronic structure results presented in this work clearly describe the behaviour of the hydrogen storage capacity and stability of Pd_1−*x*_Pt_*x*_ solid-solution NPs, based on hydrogen pressure-composition-temperature isotherm analysis.

The correlation between the hydrogen storage properties and atomic-scale/electronic structure of the Pd_1−*x*_Pt_*x*_ solid-solution NPs was investigated by synchrotron high-energy XRD coupled with PDF and RMC modelling method, EXAFS, XANES, and HAXPES. Our results demonstrate the following four findings: (1) Enhanced hydrogen storage capacity in Pd_0.92_Pt_0.08_ solid-solution NPs is connected to cavity volume of NPs. The cavity may be possible hydrogen trapping sites. (2) The observed high Pd-Pd coordination numbers of the Pd_0.92_Pt_0.08_ solid-solution NPs may also be supported to the higher hydrogen storage capacity of NPs with decreasing Pt content $x$. (3) From XANES spectra of Pd ${L}_{III}$ and Pt ${L}_{III}$-edges the hydrogen storage properties of Pd_1−*x*_Pt_*x*_ solid-solution NPs decrease with increasing Pt content owing to lower number of holes in the CB. (4) The hydride stability properties of Pd_1−*x*_Pt_*x*_ solid-solution NPs was found to be increase with decreasing Pt content, which may be explained by the upshift of the $d$-band centre closer to the Fermi level of HAXPES VB spectra and decrease of exothermic heat (${\rm{\Delta }}H$) obtained from PC isotherms. These 3D atomic-scale structure and electronic structural results reveal that the enhanced hydrogen storage capacity and stability of Pd_1−*x*_Pt_*x*_ solid-solution NPs with $0.08\le x\le 0.21$, provide an attractive strategy for further development of highly-active nanostructured materials for future energy applications.

## Methods

### Sample Preparation

Poly(*N*-vinyl-2-pyrrolidone) (PVP)-protected bimetallic Pd/Pt core/shell NPs were prepared by the combination of stepwise growth^52^ and hydrogen sacrificial protection methods^53^. The Pd_1−*x*_Pt_*x*_ solid-solution NPs were obtained by a process of hydrogen absorption/desorption (PHAD) as a trigger for the formation of Pd/Pt core/shell NPs, with a maintained size and chemical composition^5^.

### Hydride characterization

The hydrogen absorption-desorption behaviour of the Pd_1−*x*_Pt_*x*_ solid-solution NPs was measured at hydrogen pressures ranging from 10^−3^ to 101.3 kPa $via$ a volumetric technique with a pressure-composition-temperature apparatus at 303 K (Suzuki Shokan Co., Ltd., Japan).

### Structure properties

#### High-energy X-ray diffraction

High-energy X-ray diffraction data were obtained by a two-axis diffractometer available at the beamline BL04B2 in the third-generation synchrotron radiation facility at the Japan Synchrotron Radiation Research Institute (JASRI/SPring-8), Japan^54^. The incident synchrotron X-rays of 61.46 keV with a wavelength of 20.2 pm were monochromatized using an Si(220) monochromator^32^. The high-energy X-ray diffraction data of the Pd_1−*x*_Pt_*x*_ solid-solution NPs were used after background, polarization and absorption corrections.

#### Pair distribution function (PDF)

The atomic-scale structure of the Pd_1−*x*_Pt_*x*_ solid-solution NPs can also be described in terms of the total structure factor *S*(*Q*) and the pair distribution function (PDF) $g(r)$. The *S*(*Q*) is related to the coherent part, ${I}^{coh}$($Q$)^55^, of the diffraction data, given as:

3 $$S(Q)=1+\frac{[{I}^{coh}(Q)-\sum {c}_{i}|{f}_{i}(Q{)|}^{2}]}{\sum |{c}_{i}{f}_{i}(Q{)|}^{2}},$$

where ${c}_{i}$ and ${f}_{i}$ are the atomic concentration and X-ray atomic scattering factor, respectively, for the atomic species of type $i$. The $S(Q)$ over a wide range of $Q$ enables a higher resolution to be obtained in the real-space information $g(r)$ using

4 $$g(r)=1+\frac{1}{2{\pi }^{2}r\rho }{\int }_{{Q}_{min}}^{{Q}_{max}}Q[S(Q)-\mathrm{1]}sin(Qr)dQ,$$

where $\rho$ is the atomic number density and $r$ is the radial distance. The reduced PDF, *G*(*r*), defined by *G*(*r*) = 4*πrρ*(*g*(*r*) - 1). Instead of the *g*(*r*) and *G*(*r*), the total correlation function *T*(*r*) is also used for data plotting. This is defined as *T*(*r*) = 4*πrρg*(*r*).

#### Reverse Monte Carlo (RMC) modelling

The RMC models were performed on the Pd_1−*x*_Pt_*x*_ solid-solution NPs for $0.08\le x\le 0.21$ with the use of the RMC_POT software^56^ furnished for the case of non-periodic boundary conditions. The RMC runs were performed for the Pd_1−*x*_Pt_*x*_ solid-solution NPs, which closely resemble the spherical shape configurations. The RMC models were guided by the experimental structure factor and were stopped when the computer-calculated and experimental *S*(*Q*) data were perfectly aligned over the entire range of wave vectors.

### X-ray absorption fine structure (XAFS)

The XAFS measurements of the Pt ${L}_{III}$-edge (11.56 keV) and Pd $K$-edge (24.35 keV) were performed using Si(111) and Si(311) monochromator crystals, respectively, in the BL01B1 beamline at the JASRI/SPring-8, Japan. All XAFS spectra were recorded in transmission mode at room temperature. The Pd_1−*x*_Pt_*x*_ solid-solution NP samples were placed between two pieces of Kapton tapes, which sandwiched a 0.5 mm-thick copper spacer featuring a hole with a 3.0 mm inner diameter. The intensities of the incident and transmitted X-ray beams were monitored with ionization chambers. To extract the XAFS oscillation, $\chi (k)$ (where $k$ is the wave number of the photoelectron) from the observed absorption spectrum, the extrapolated pre-edge background absorption was subtracted first. The resulting curve was then normalized by dividing the jump of the absorption at the edge. The EXAFS analysis was performed using the Athena and Artemis program in the IFEFFIT software package^57^.

The XAFS measurements on the Pd ${L}_{III}$-edge were performed with a Si(111) double-crystal monochromator at BL06 in the Kyushu synchrotron light research centre (SAGA-LS), Japan. Spectra of the Pd ${L}_{III}$-edge XANES at 3.17 keV were acquired in fluorescence mode at room temperature. The Pd-${L}_{\alpha }$ fluorescence emission yield was collected with a silicon drift detector. Specimens were diluted by high-purity hexagonal BN powder before measurement, and intensities of the XANES spectra were normalized using the post-edge higher energy region.

### Hard X-ray photoelectron spectroscopy (HAXPES)

The HAXPES experiments were performed with synchrotron radiation linearly polarized in the horizontal plane with a photon energy of 5.95 keV using a Si (111) double-crystal monochromator and the Si (333) reflection of a channel-cut monochromator, located at the National Institute for Materials Science (NIMS) contract undulator beamline BL15XU of JASRI/SPring-8, Japan^58^. The photoelectrons were collected and analysed using a high-resolution hemispherical analyser (VG Scienta R4000, Sweden), which was set so that the angle between the X-ray direction and the path of the photoelectrons entering the analyser was $90$^ °59^. The total energy resolution was estimated to be 240 meV, which was confirmed by measuring the Fermi cut-off of an evaporated Au film. The binding energy was referenced to the Fermi level (E_*F*_) of Au, and the experiments were performed at room temperature with a take-off angle of 88° with respect to the sample plate surface. The photoelectron inelastic mean free paths of the Pd and Pt bulk materials were approximately 5.7 and 4.8 nm, respectively, according to the TPP-2M formula^60^. The effective photoelectron information is found in the range of about 15–17 nm, which is greater than the diameter of the largest NP. Hence, we were able to obtain information regarding the electronic structures of the Pd_1−*x*_Pt_*x*_ solid-solution NPs for $0\le x\le 0.5$.

### Data availability

The authors declare that the data supporting the findings of this study are available within the paper and its Supplementary Information file and from the corresponding author upon reasonable request.

## Electronic supplementary material

Supplementary Information
