# Publisher Correction: Hydrogen storage and stability properties of Pd–Pt solid-solution nanoparticles revealed *via* atomic and electronic structure

**DOI:** 10.1038/s41598-018-20337-w

**Published:** 2018-01-23

**Authors:** Loku Singgappulige Rosantha Kumara, Osami Sakata, Hirokazu Kobayashi, Chulho Song, Shinji Kohara, Toshiaki Ina, Toshiki Yoshimoto, Satoru Yoshioka, Syo Matsumura, Hiroshi Kitagawa

**Affiliations:** 1 Synchrotron X-ray Station at SPring-8, Research Network and Facility Services Division, National Institute for Materials Science (NIMS), 1-1-1 Kouto, Sayo-cho, Sayo-gun, Hyogo 679-5148 Japan; 2 Synchrotron X-ray Group, Research Center for Advanced Measurement and Characterization, NIMS, 1-1-1 Kouto, Sayo-cho, Sayo-gun, Hyogo 679-5148 Japan; 3 Department of Materials Science and Engineering, School of Materials and Chemical Technology, Tokyo Institute of Technology, 4259-J3-16, Nagatsuta-cho, Midori-ku, Yokohama 226-8502 Japan; 4 Division of Chemistry, Graduate School of Science, Kyoto University, Kitashirakawa Oiwake-cho, Sakyo-ku, Kyoto 606-8502 Japan; 5 Research & Utilization Division, Japan Synchrotron Radiation Research Institute (JASRI), 1-1-1 Kouto, Sayo-cho, Sayo-gun, Hyogo 679-5198 Japan; 6 Department of Applied Quantum Physics and Nuclear Engineering, Kyushu University, 744 Motooka, Nishi-ku, Fukuoka 819-0395 Japan; 7 INAMORI Frontier Research Center, Kyushu University, 744 Motooka, Nishi-ku, Fukuoka 819-0395 Japan; 8 Institute for Integrated Cell-Material Sciences (iCeMS), Kyoto University, Yoshida, Sakyo-ku, Kyoto 606-8501 Japan

Correction to: *Scientific Reports* 10.1038/s41598-017-14494-7, published online 03 November 2017

The original HTML version of this Article contained a typographical error in the publication date ‘03 November 2017’ which was incorrectly given as ‘06 November 2017’. This has now been corrected in the HTML version of the Article.

