# Supplementary material for: Hydrogen storage and stability properties of Pd–Pt solid-solution nanoparticles revealed via atomic and electronic structure
Source: Sci Rep. 2017 Nov 3;7:14606. doi: 10.1038/s41598-017-14494-7 (PMC5668347; doi:10.1038/s41598-017-14494-7)
Supplement: Supplementary file 1 — Supplementary Information [file 41598_2017_14494_MOESM1_ESM.pdf]

Supplementary Information

Hydrogen storage and stability properties of Pd–Pt solid-solution nanoparticles revealed *via* atomic and electronic structure

Loku Singgappulige Rosantha Kumara [1,*], Osami Sakata[1,2,3,*], Hirokazu Kobayashi[4], Chulho Song[1], Shinji Kohara[1,2], Toshiaki Ina[5], Toshiki Yoshimoto[6], Satoru Yoshioka[6], Syo Matsumura[6,7] & Hiroshi Kitagawa[4,7,8]

[1]*Synchrotron X-ray Station at SPring-8, Research Network and Facility Services Division, National Institute for Materials Science (NIMS), 1-1-1 Kouto, Sayo-cho, Sayo-gun, Hyogo 679-5148, Japan.*

[2]*Synchrotron X-ray Group, Research Center for Advanced Measurement and Characterization, NIMS, 1-1-1 Kouto, Sayo-cho, Sayo-gun, Hyogo 679-5148, Japan.*

[3]*Department of Materials Science and Engineering, School of Materials and Chemical Technology, Tokyo Institute of Technology, 4259-J3-16, Nagatsuta-cho, Midori-ku, Yokohama 226-8502, Japan.*

[4]*Division of Chemistry, Graduate School of Science, Kyoto University, Kitashirakawa Oiwake-cho, Sakyo-ku, Kyoto 606-8502, Japan.*

[5]*Research & Utilization Division, Japan Synchrotron Radiation Research Institute (JASRI), 1-1-1 Kouto, Sayo-cho, Sayo-gun, Hyogo 679-5198, Japan.*

[6]*Department of Applied Quantum Physics and Nuclear Engineering, Kyushu University, 744 Motooka, Nishi-ku, Fukuoka 819-0395, Japan.*

[7]*INAMORI Frontier Research Center, Kyushu University, 744 Motooka, Nishi-ku, Fukuoka 819-0395, Japan.*

[8]*Institute for Integrated Cell-Material Sciences (iCeMS), Kyoto University, Yoshida, Sakyo-ku, Kyoto 606-8501, Japan.*

*Corresponding authors: L. S. R. Kumara, E-mail: KUMARA.Rosantha@nims.go.jp
O. Sakata, E-mail: SAKATA.Osami@nims.go.jp

S1. Nanoparticles size and composition analysis

Transmission Electron Microscopy (TEM) analysis

The size of the prepared samples was determined from transmission electron microscopy (TEM) images, which were obtained using a Hitachi HT7700 transmission electron microscope operated at 100 kV accelerate voltage. The samples dispersed with ethonal were drop-cast onto a carbon-coated copper grid and allowed to dry under ambient conditions. The mean diameter and distributions were estimated by averaging over 200 particles. The average particle sizes of the $Pd_{1-x}Pt_x$ solid-solution NPs for compositions where x = 0, 0.08, 0.15, 0.21, and 0.5 are 6.1 ± 0.8, 6.7 ± 0.9, 7.4 ± 0.9, 8.1 ± 1.0, and 11.2 ± 1.7 nm, respectively[1].

Inductively coupled plasma mass spectrometry (ICP-MS) analysis

The atomic composition of Pt in the $Pd_{1-x}Pt_x$ solid-solution NPs were estimated to be 8, 15, 21 and 50% with errors smaller than 1% by inductively coupled plasma mass spectrometry (ICP-MS)[1].

S2. High-energy X-ray diffraction data analysis

X-ray diffraction measurements were performed using a two-axis diffractometer installed at the BL04B2 beamline[2] of the third-generation synchrotron radiation facility SPring-8, Hyogo, Japan. The incident X-ray beam was 61.46 keV, with a wavelength of 0.02019 nm; it was generated using an Si(220) monochromator. The $Pd_{1-x}Pt_x$ solid-solution and core/shell NPs were loaded into a capillary column and measured at room temperature. Fine powders of bulk 99.9% Pd and Pt were used as the reference materials. The XRD data were corrected for background, polarization and absorption. As shown in Figs. 1 and S1, the corrected XRD data were then normalized to the structure factor $S(Q)$ and Fourier transformed to produce the pair distribution function $g(r)$ data using the SPring-8 BL04B2 PDF analysis software.

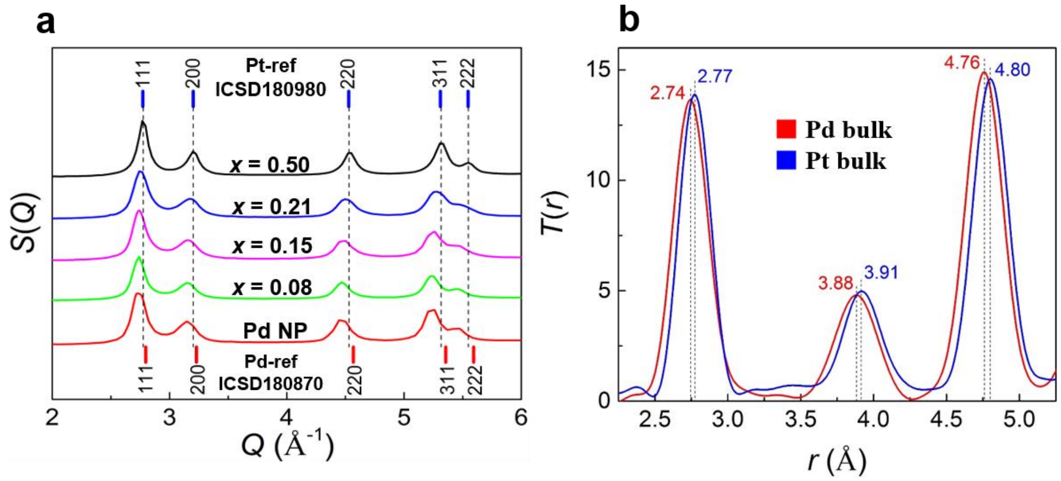

Figure S1: High-energy X-ray diffraction (XRD) data. High-energy XRD patterns in total structure factor, $S(Q)$ and total correlation function, $T(r)$. (a) $S(Q)$ of $Pd_{1-x}Pt_x$ (Pd/Pt) core/shell NPs, and (b) $T(r)$ of fcc-type Pd and Pt bulk metals.

S3. Pair distribution function (PDF) analysis

The atomic scale structure of a $Pd_{1-x}Pt_x$ solid-solution NPs can be described quantitatively in terms of pair distribution function (PDF), $g(r)$, which indicates the average probability of finding another atom within a specified volume at a distance from an origin atom as a function of the radial distance r.[3] This is Fourier transform of the total structure factor $S(Q)$, defined by following equation:

$$g(r) = 1 + \frac{1}{2\pi^2 r\rho}\int_{Q_{min}}^{Q_{max}} Q[S(Q) - 1]\sin(Qr)\, dQ,$$

where ρ is the atomic number density and r is the radial distance. The diffraction wave vector, Q, is defined by $Q = 4\pi\, sin\theta/\lambda$, where θ is half the scattering angle and λ is the wavelength of the incident X-rays.

Instead of the $g(r)$, another widely used correlation function is the reduced pair distribution function, $G(r)$, defined by

$$G(r) = 4\pi r\rho(g(r) - 1).$$

The main advantage of $G(r)$ function is the one directly obtained from the Fourier transform of total structure factor $S(Q)$ without knowing average number density ρ of the materials.[4] The other useful correlation function is radial distribution function (RDF) is given by

$$RDF = 4\pi r^2 \rho g(r).$$

At the large values of r, the RDF tends to the smooth parabolic function $4\pi r^2\rho$. On the other hand, the RDF should be equal to zero at small values of r. The area under the respective peak in the RDF equal to the number of neighbors to the origin atom (*i.e.* the average coordination number). The total correlation function, $T(r)$, is also used for data plotting. This is defined by

$$T(r) = 4\pi r\rho g(r).$$

The $T(r)$ also known as reduced RDF, which is easily obtained by dividing the RDF by r and improve the precision of peaks for better estimation of average coordination number.

Crystal structural information of $Pd_{1-x}Pt_x$ solid-solution NPs were obtained by structural refinement of reduced PDF $G(r)$ data using PDFgui software package[5]. The parameter qdamp was obtained by refining a known CeO_2 NIST standard. Then the qdamp parameter was fixed for PDF refinement of all NPs and bulk data. The calculated model and experimental $G(r)$ data with the difference curve of $Pd_{1-x}Pt_x$ solid solution NPs for $0.08 \leq x \leq 0.5$ are shown in Fig. S2. The refined residual (R_w) values obtained by refinement are 0.16, 0.17, 0.15 and 0.17 for $Pd_{0.5}Pt_{0.5}$, $Pd_{0.79}Pt_{0.21}$, $Pd_{0.85}Pt_{0.15}$, and $Pd_{0.92}Pt_{0.08}$ solid-solution NPs, respectively.

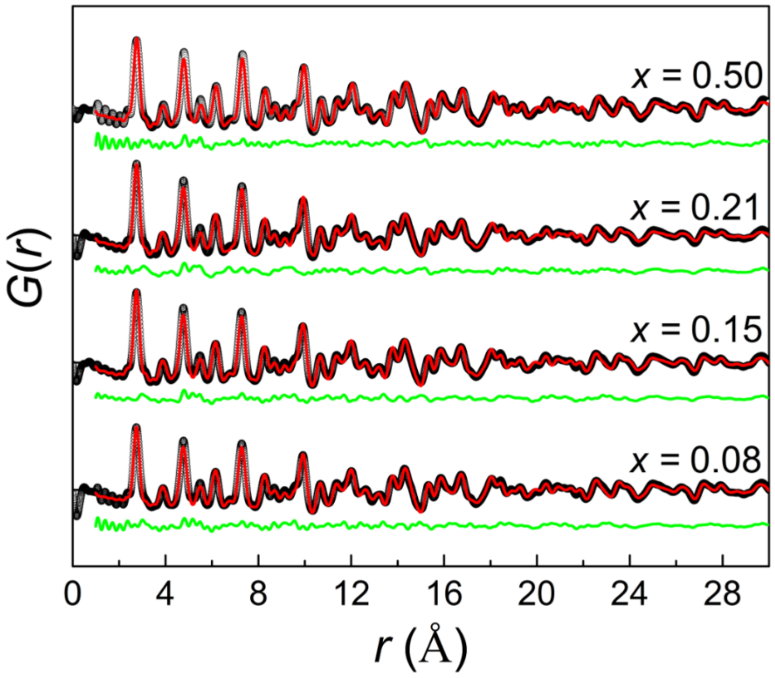

Figure S2: PDF fits to $Pd_{1-x}Pt_x$ NPs data. The experimental $G(r)$ (black open circle), the calculated $G(r)$ (red solid line), and their difference curves (green solid line) from the PDFgui refined structural model of $Pd_{1-x}Pt_x$ solid solution NPs.

S4. Reverse Monte Carlo (RMC) simulation

The 3D structural models of $Pd_{1-x}Pt_x$ solid-solution NPs were generated by RMC modeling method using RMC_POT software[6] furnished for the case of non-periodic boundary conditions. RMC model of $Pd_{0.79}Pt_{0.21}$ solid-solution NPs was constructed by 13815 (Pd; 10914, Pt; 2901) total atoms in a spherical configuration closely resembling that of a spherical nanoparticles with 8.1 nm in diameter and 0.0496461 $Å^{-3}$ in number density. As shown in Figure 3, the calculated total structure factor $S(Q)$ and experimental data shows good agreement for $Pd_{0.79}Pt_{0.21}$,$Pd_{0.85}Pt_{0.15}$, and $Pd_{0.92}Pt_{0.08}$ solid-solution NPs.

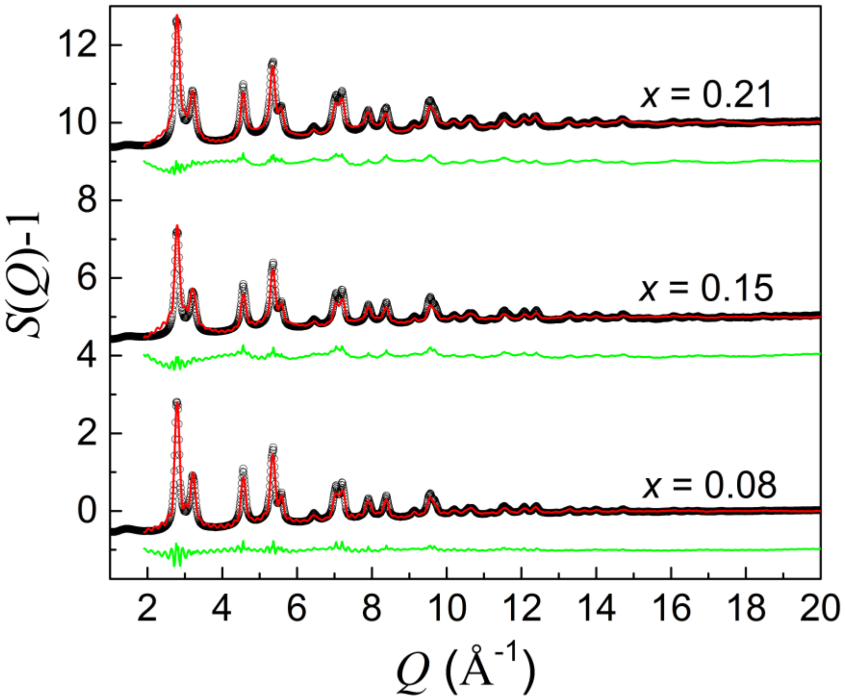

Figure S3: Experimental and RMC generated X-ray total structure factor *S*(*Q*)-1 for $Pd_{0.79}Pt_{0.21}$, $Pd_{0.85}Pt_{0.15}$, and $Pd_{0.92}Pt_{0.08}$ solid-solution NPs. The experimental and RMC generated total structure factor *S*(*Q*)-1 data are shown as black open sphere and red solid line, respectively. The green solid line represent the differences of experimental and RMC generated *S*(*Q*)-1 for each solid-solution NPs with off curves.

S5. X-ray absorption fine structure (XAFS) analysis

The XAFS experiment was performed at the Pt L_{III}-edge and Pd *K*-edge in transmission mode at room temperature. Figure S4 show the X-ray absorption near-edge structure (XANES) spectra at Pd *K*-edge for Pd-Pt solid solution NPs and Pd metal foil. The extended X-ray absorption fine structure (EXAFS) spectra at the Pt L_{III}-edge and Pd *K*-edge were Fourier-transformed to real space (*R*-space) in the *k* range 3.0 to 15.0 Å^{-1}. Figure S6 show the Fourier transformations of Pt L_{III}-edge k^3-weighted EXAFS spectra (phase shift uncorrected) of $Pd_{1-x}Pt_x$ solid solution NPs and Pt metal foil. The Pd *K*-edge EXAFS fit was performed in *R*-space using Artemis program in the IFEFFIT[7]. Here the single scattering path between the Pd core atom and its nearest neighbor was fit with k^3-weight. The results of Pd-*K* edge EXAFS refinement of $Pd_{1-x}Pt_x$ solid-solution NPs are reported in the Table S1.

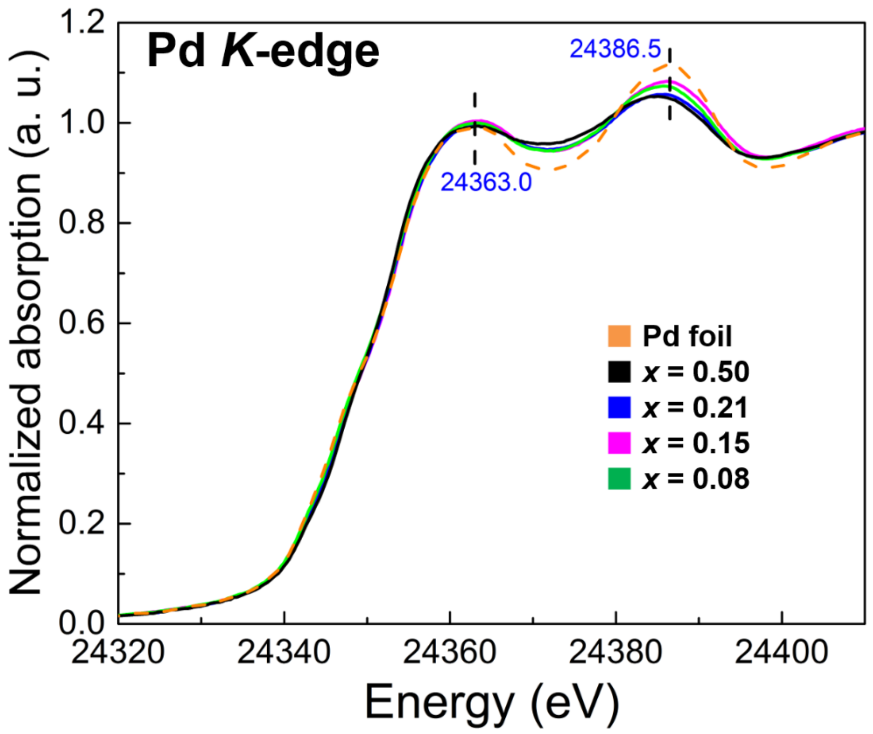

Figure S4: XANES spectra at Pd *K*-edge. The Pd *K*-edge XANES spectra of $Pd_{1-x}Pt_x$ solid solution NPs and Pd metal foil.

S6. X-ray absorption near-edge structure (XANES) of Pd L_{III}-edge

As shown in Figure S5a, the Pd L_{III}-edge XANES spectra of $Pd_{1-x}Pt_x$ solid-solution NPs and Pd metal foil were obtained by fluorescence mode at room temperature. The L_{III}-edge shows strong peak intensity at the top of absorption edge, also known as "white line" indicate unoccupied *d*-band states above the Fermi-level[8]. This white line of Pd L_{III}-edge corresponds to the $2p_{3/2} \rightarrow 4d$ electronic transitions. The integrated white line intensity was evaluated by integrating the area from 3160 to 3190 eV, which is proportional to the number of 4*d*-band vacancies (holes) in the NPs. The correlation between the integrated white line intensity and hydrogen storage capacity is shown in Fig. 5b. In addition, Fig. 5c shows the peak maxima (excitation energy) of the white line as a function of Pd content of $Pd_{1-x}Pt_x$ solid-solution NPs.

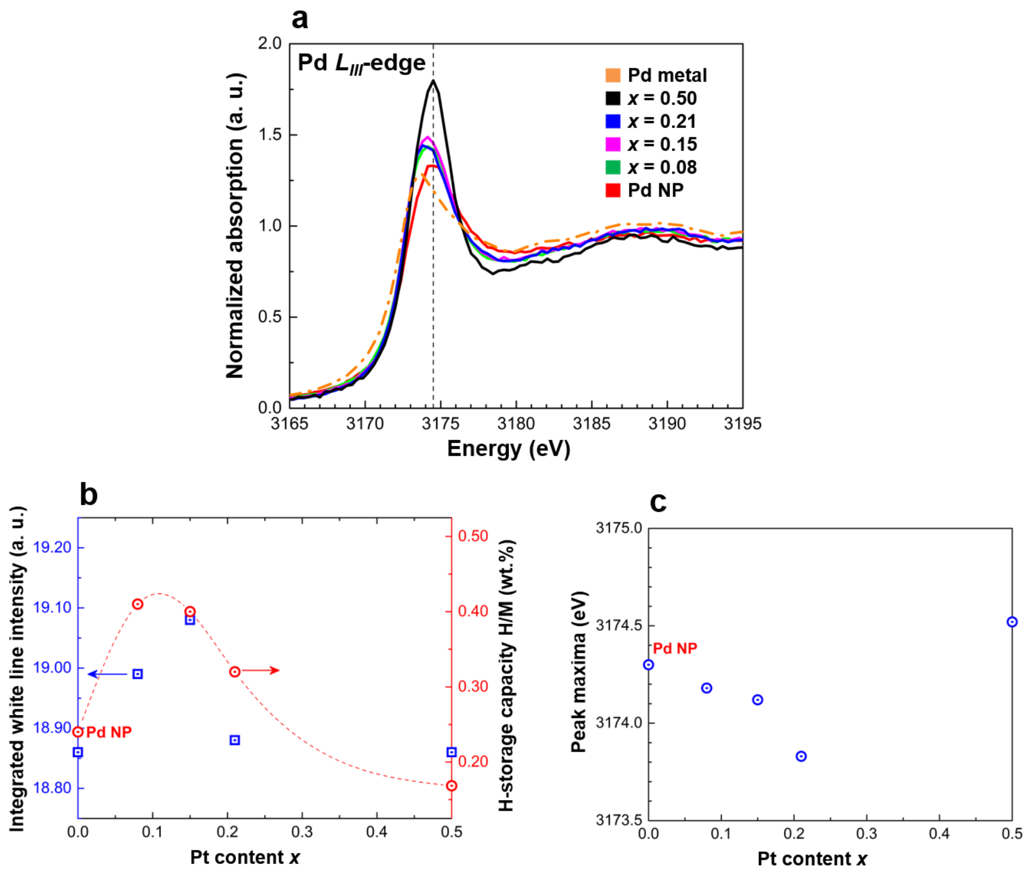

Figure S5: Pd L_{III}-edge XANES analyses. (a) A comparison of XANES spectra at the Pd L_{III}-edge for $\mathbf{Pd_{1-x}Pt_x}$ solid solution NPs and Pd metal powder. (b) The integrated white line intensity (blue square) and hydrogen storage capacity H/M (red circle) vs Pd composition. (c) The peak maxima (excitation energy) of XAFS data for $Pd_{1-x}Pt_x$ solid solution NPs.

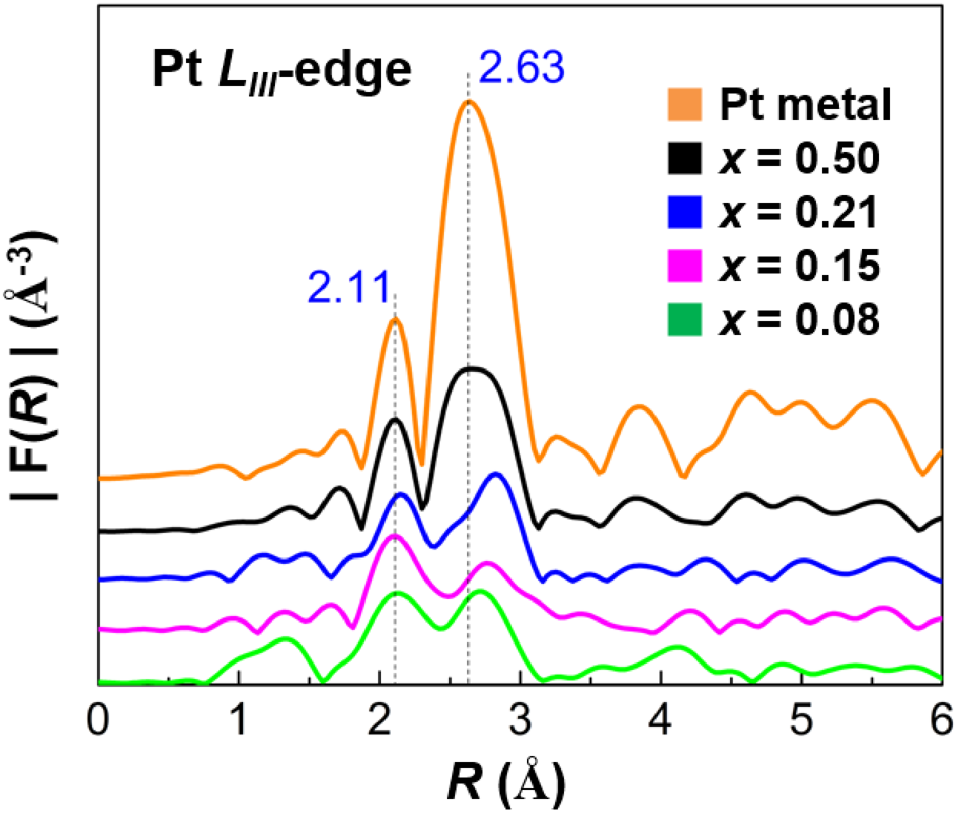

Figure S6: EXAFS analysis at Pt L_{III}-edge. Fourier transformations Pt L_{III}-edge EXAFS spectra (phase shift uncorrected) of $Pd_{1-x}Pt_x$ solid solution NPs and Pt metal foil.

Table S1: EXAFS fittings data. Pd *K*-edge EXAFS fittings data of bimetallic $Pd_{1-x}Pt_x$ solid solution NPs, monometallic Pd NPs, and Pd foil.

Sample	R_{1NN} (Å)	N	σ^2 (10^{-3} Å^2)	R factor (%)
$Pd_{0.5}Pt_{0.5}$ NPs	2.74 ± 0.01	6.8 ± 0.9	6.9 ± 0.6	0.008
$Pd_{0.79}Pt_{0.21}$ NPs	2.74 ± 0.02	8.0 ± 0.8	7.1 ± 0.4	0.004
$Pd_{0.85}Pt_{0.15}$ NPs	2.74± 0.02	9.7 ± 0.7	6.9 ± 0.3	0.002
$Pd_{0.92}Pt_{0.08}$ NPs	2.74± 0.02	9.4 ± 0.6	7.1 ± 0.3	0.001
Pd NPs	2.77± 0.01	7.3 ± 0.6	8.4 ± 0.6	0.004
Pd bulk	2.74 ± 0.01	12	5.8 ± 0.3	0.002

S7. Pressure-composition-temperature isotherms measurement

As shown in Figure 6 in main text, the hydrogen pressure-composition-temperature (PCT) isotherms were measured at 303 K for $Pd_{0.5}Pt_{0.5}$, $Pd_{0.79}Pt_{0.21}$, $Pd_{0.85}Pt_{0.15}$, and $Pd_{0.92}Pt_{0.08}$ solid-solution NPs by a volumetric technique using PCT apparatuses. The $Pd_{1-x}Pt_x$ solid-solution NPs shows very clear plateau-like region compared to Pd/Pt core/shell NPs[1].

References

[S1]. Kobayashi, H. *et al*. Atomic-Level Pd-Pt Alloying and Largely Enhanced Hydrogen-Storage Capacity in Bimetallic Nanoparticles Reconstructed from Core/Shell Structure by a Process of Hydrogen Absorption/Desorption. *J. Am. Chem. Soc.* **132**, 5576–5577 (2010).

[S2]. Kohara, S. *et al.* Structural Studies of Disordered Materials using High-Energy X-ray Diffraction from Ambient to Extreme Conditions. *J. Phys.: Condens. Matter* **19**, 506101–506115 (2007).

[S3]. Waseda, Y. Anomalous X-Ray Scattering for Materials Characterization: Atomic-Scale Structure Determination 9–13, (Springer, 2003).

[S4]. Egami, T. & Billinge, S. J. L. Underneath the Bragg Peaks Structural Analysis of Complex Materials 87–90, (Elsevier, 2012).

[S5]. Farrow, C. L. *et al.* PDFfit2 and PDFgui: computer programs for studying nanostructure in crystals. *J. Phys.: Condens. Matter* **19**, 335219 (2007).

[S6]. Gereben, O. & Petkov, V. Reverse Monte Carlo Study of Spherical Sample Under Non-Periodic Boundary Conditions: The Structure of Ru Nanoparticles Based on X-ray Diffraction Data. *J. Phys.: Condens. Matter* **25**, 454211–454219 (2013).

[S7]. Ravel, B. & Newville, M. *ATHENA*, *ARTEMIS*, *HEPHAESTUS*: data analysis for X-ray absorption spectroscopy using IFEFFIT. *J. Synchrotron Radiat.* **12**, 537–541 (2005).

[S8]. Clancy, J. P. *et al*. Spin-orbit coupling in iridium-based 5*d* compounds probed by x-ray absorption spectroscopy. Phys. Rev.B **86**, 195131 (2012).
